# Complementary roles of standing, supine, and valgus-stress whole-leg radiographs in medial open-wedge high tibial osteotomy planning: supine minimizes bias, whereas standing and valgus-stress reduce outliers

**DOI:** 10.1186/s43019-026-00340-6

**Published:** 2026-08-06

**Authors:** Geunwu Gimm, Hyunjeong Ra, Seok Gyun Yang, Byung Sun Choi, Du Hyun Ro, Hyuk-Soo Han

**Affiliations:** 1 Department of Biomedical Engineering, Seoul National University College of Medicine, Seoul, Republic of Korea; 2 Department of Orthopaedic Surgery, Seoul National University Hospital, Seoul, Republic of Korea; 3 Seoul National University College of Medicine, Seoul, Republic of Korea; 4 Department of Orthopaedic Surgery, Seoul National University College of Medicine, Seoul, Republic of Korea; 5 CONNECTEVE Co., Ltd, Seoul, Republic of Korea

**Keywords:** High tibial osteotomy, Preoperative planning, Whole-leg radiograph, Standing, Supine, Valgus-stress

## Abstract

**Background:**

Accurate alignment correction is essential in medial open-wedge high tibial osteotomy (MOWHTO). However, conventional planning based on standing radiographs may not reflect postoperative intra-articular alignment changes. While differences between standing and supine radiographs have been investigated, the role of valgus-stress whole-leg radiographs in preoperative planning remains largely unexplored. This study evaluated the predictive accuracy of standing, supine, and valgus-stress whole-leg radiographs in MOWHTO planning and determined whether integrating these modalities improves alignment prediction compared with single-modality planning.

**Methods:**

This retrospective study included 71 patients who underwent MOWHTO. Preoperative standing, supine, and valgus-stress whole-leg radiographs, along with 1-year postoperative standing radiographs, were analyzed. To minimize the influence of surgical execution error and allow the analysis to focus on planning error, the achieved wedge angle was back-calculated from postoperative bony correction and reapplied to each preoperative modality to simulate the postoperative weight-bearing line ratio (Post-WBLR). Accuracy was assessed by comparing simulated and actual Post-WBLR. Multivariable regression was performed to identify predictors of actual Post-WBLR and to compare a multimodal model with the best-performing single-modality simulation.

**Results:**

In the postoperative valgus subgroup (*n* = 48), supine-based simulation showed no significant difference from actual Post-WBLR (mean difference, 0.0% ± 6.9%; root mean square error [RMSE], 6.8%). In contrast, standing-based simulation underestimated alignment (−9.3% ± 7.8%; RMSE, 12.1%), whereas valgus-stress simulation overestimated it (15.2% ± 6.7%; RMSE, 16.6%). A multimodal regression model incorporating simulated Post-WBLR from standing and valgus-stress radiographs demonstrated significantly improved predictive accuracy compared with the supine-only simulation (*R*^2^, 0.606 versus 0.080; RMSE, 4.5% versus 6.8%). Notably, in patients with a preoperative standing-to-supine joint line convergence angle difference (∆JLCA_standing–supine_) ≥ 2.7°, the multimodal model significantly reduced prediction errors (absolute error: 3.2% versus 6.8%; RMSE: 4.0% versus 9.0%; all *P* < 0.015).

**Conclusions:**

Supine radiographs generally provided the most accurate single-modality basis for MOWHTO planning. Standing and valgus-stress radiographs offered complementary information, and their integration improved prediction accuracy and reduced planning outliers. While further validation is required, a stepwise planning workflow—using supine radiographs as the default modality and multimodal integration when ∆JLCA_standing–supine_ ≥ 2.7°—may serve as an exploratory framework for reducing the risk of planning-related under- or overcorrection.

**Supplementary Information:**

The online version contains supplementary material available at 10.1186/s43019-026-00340-6.

## Background

Medial open-wedge high tibial osteotomy (MOWHTO) is a well-established treatment for medial unicompartmental knee osteoarthritis with varus deformity, particularly in middle-aged patients [1]. By correcting the alignment to a slight valgus position, the procedure effectively redistributes joint loading from the degenerated medial side to the relatively preserved lateral compartment [2]. Achieving accurate postoperative alignment is crucial. Undercorrection can lead to persistent varus and treatment failure, while overcorrection may cause lateral compartment osteoarthritis and result in poor clinical outcomes along with cosmetic issues [2–5].

The pursuit of precision in MOWHTO has evolved from a simple focus on bony correction to a comprehensive understanding of dynamic intra-articular behaviors, as lower limb alignment is influenced by both bony and intra-articular components. While traditional planning methods based on standing radiographs, such as those proposed by Miniaci [6] and Dugdale [7], have provided a widely used framework, they often fail to achieve the desired alignments. Miniaci himself reported that only 50% of osteotomies fell within a satisfactory range (±10%) [8], and this limited accuracy may reflect not only surgical execution error but also limitations of correction planning based solely on bony calculations.

With advances in technologies such as navigation and patient-specific instrumentation decreasing surgical execution errors [9], the focus has shifted to planning errors—particularly those related to intra-articular alignment corrections caused by soft-tissue laxity, as indicated by changes in the joint line convergence angle (JLCA). Several studies have examined the differences in JLCA between standing and supine radiographs, as well as the variations in planning results derived from these two imaging modalities [10–16].

From a biomechanical perspective, shifting the weight-bearing line in the direction of valgus alignment may introduce a torque that produces valgus stress at the joint. Additionally, the tension in ligaments varies with alignment: lateral structures are under tension in a varus position, while medial structures are tense in a valgus position. Therefore, relying solely on varus standing and supine radiographs may not fully replicate the biomechanical environment around the knee after it has been corrected to a valgus alignment. In this context, valgus-stress radiographs may provide a biomechanically plausible approximation of postoperative medial soft-tissue tension after valgus correction. Previous studies have examined valgus-stress radiography in related contexts, including JLCA, articular gap, and soft-tissue laxity on localized knee radiographs [4, 17, 20 –]. Nevertheless, valgus-stress whole-leg radiographs have rarely been evaluated as a modality for preoperative alignment simulation or planning in MOWHTO.

This study aimed to evaluate the effectiveness of standing, supine, and valgus-stress whole-leg radiographs in preoperative planning for MOWHTO. We examined how accurately each radiographic modality simulates postoperative standing alignment and whether combining simulations from multiple modalities enhances prediction accuracy. We hypothesized that supine-based planning would minimize systematic bias, while standing and valgus-stress radiographs would provide complementary information, improving prediction accuracy and decreasing planning outliers when used together.

## Methods

### Patient inclusion

This retrospective study received approval from institutional review board of our hospital. A total of 79 consecutive patients who underwent MOWHTO for symptomatic medial compartment osteoarthritis between November 2019 and February 2025 were included. The exclusion criteria were: (1) absence of preoperative supine or valgus-stress whole-leg radiographs (*n* = 5); (2) lack of follow-up standing whole-leg radiographs between 6 months and 2 years postoperatively (*n* = 2); (3) presence of congenital knee abnormalities or anomalies (*n* = 1); and (4) inability to undergo valgus-stress examination due to severe pain, instability, or noncooperation/noncompliance (*n* = 0). Consequently, 71 patients were included in the final analysis.

### Surgical technique

All surgical procedures were conducted by four experienced knee surgeons at a single tertiary center utilizing a standardized technique. A longitudinal incision was made along the medial aspect of the proximal tibia. The pes anserinus was retracted, and the superficial medial collateral ligament (sMCL) was elevated subperiosteally and retracted posteriorly. Under fluoroscopic guidance, two Kirschner wires were inserted at the medial metaphyseal–diaphyseal junction (approximately 3.5–4.0 cm distal to the joint line) and advanced toward the lateral hinge point between the tip of the fibular head and the lateral tibial cortex. A biplanar osteotomy was performed, which included a horizontal osteotomy along the Kirschner wires while preserving the lateral cortex, followed by a coronal osteotomy conducted nearly parallel to the anterior tibial cortex and proximal to the horizontal osteotomy line. The osteotomy gap was gradually opened to the preoperatively planned wedge angle based on the Miniaci method [6], and the mechanical axis was assessed under fluoroscopy to confirm alignment at approximately 62.5% WBLR. The osteotomy was stabilized using a locking plate (Tomofix; DePuy Synthes).

### Radiologic assessment

Radiologic parameters were assessed using preoperative (standing, supine, and valgus-stress) and 1-year postoperative (standing) whole-leg radiographs. The weight-bearing line ratio (WBLR) and hip–knee–ankle angle (HKAA) indicate overall limb alignment involving both bony and intra-articular components. The mechanical lateral distal femoral angle (mLDFA) and medial proximal tibial angle (MPTA) represent the bony alignment of the femur and tibia, respectively. The JLCA reflects intra-articular alignment influenced by intra-articular structures and periarticular soft tissues. For HKAA, neutral alignment was defined as 180°, with values < 180° indicating a varus alignment. For JLCA, medial joint space narrowing was recorded as a positive value, whereas lateral joint space narrowing was recorded as a negative value. The joint line obliquity angle (JLOA), which requires a weight-bearing assessment, was measured on standing radiographs, with positive values indicating an apex-proximal (medial-upward) orientation. Detailed descriptions of these alignment parameters are provided in a previous study [2]. Arithmetic HKAA (aHKAA) was calculated as 180 + MPTA − mLDFA to represent the overall bony alignment of the limb, excluding the effects of intra-articular alignment on the HKAA.

**Fig. 1:**
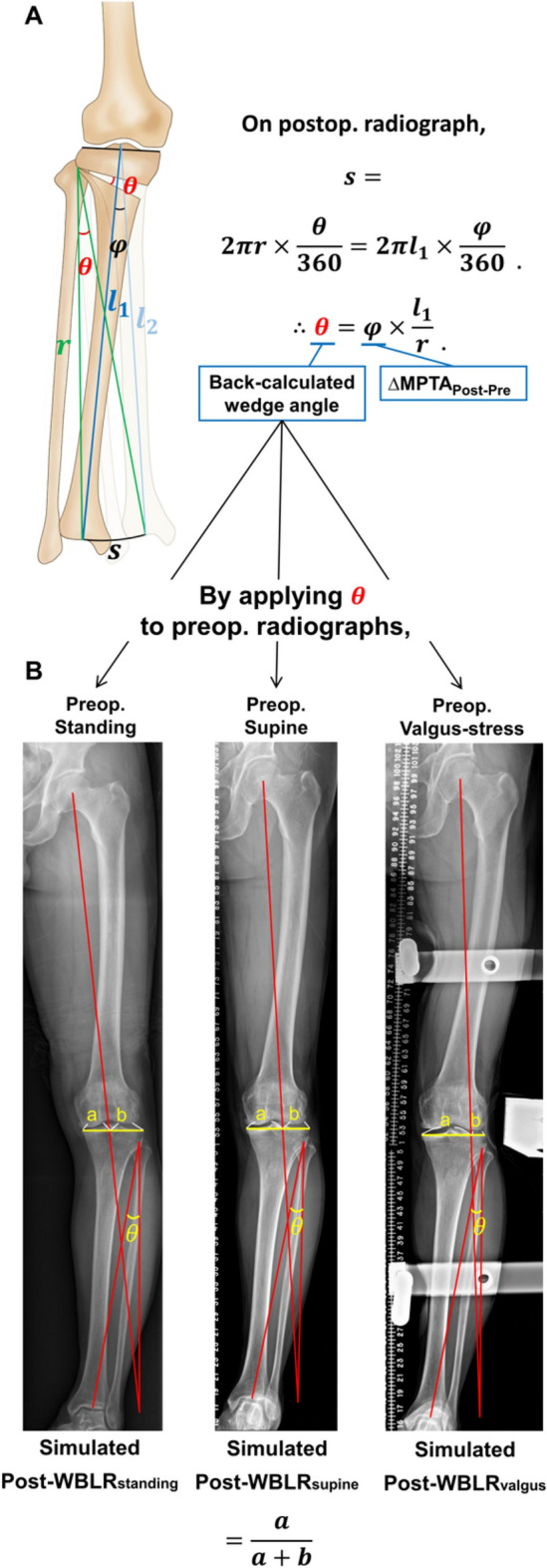
**A** Using the arc length (*s*), the achieved wedge angle (*θ*) can be back-calculated from the postoperative change in MPTA on standing radiographs (∆MPTA_Post–Pre_,* φ*). *r* represents the distance from the ankle center to the hinge point, and *l*_1_ denotes the distance from the ankle center to the center of the tibial plateau. **B** The back-calculated wedge angle is applied to preoperative standing, supine, or valgus-stress whole-leg radiographs using the Miniaci concept to simulate the Post-WBLR. MPTA, medial proximal tibial angle; Post, postoperative; Pre, preoperative; WBLR, weight-bearing line ratio

Valgus-stress whole-leg radiographs were obtained with the patient in the supine position on the radiographic table, with the knee fully extended. A valgus force of 150 N was applied at the joint line level using a Telos stress device (Telos GmbH). The Telos device operates through a paired action–reaction mechanism; accordingly, no separate ankle fixation is required (Fig. 1B). The severity of osteoarthritis was assessed using the Kellgren–Lawrence (K-L) grade on the basis of standing knee anteroposterior radiographs. All alignment parameters and K-L grades were analyzed with CONNEVO KOA V1.0.0 and ALI V1.0.0 software (CONNECTEVE Co., Ltd, South Korea), an artificial intelligence model previously validated for reliability across diverse institutional imaging protocols (interobserver intraclass correlation coefficient [ICC], 0.936–0.997) [21].

### Postoperative back-calculation of achieved wedge angle

The achieved opening wedge angle was calculated postoperatively by determining the change in MPTA between preoperative and postoperative standing radiographs (∆MPTA_Post–Pre_) and bony lengths (Fig. 1A). In principle, the overall error in achieving the target WBLR can be divided into two components: (1) preoperative planning error related to the intended wedge angle and (2) intraoperative surgical error resulting from imperfect execution of the planned correction [22]. By back-calculating the wedge angle from the achieved bony correction, surgical error was assumed to be zero. This assumption allowed the subsequent analyses to focus primarily on planning error.

### Simulation of postoperative WBLR

The back-calculated wedge angle was applied to preoperative standing, supine, and valgus-stress radiographs using the Miniaci planning concept to simulate the postoperative WBLR (Post-WBLR) (Fig. 1). Unlike conventional Miniaci planning, which determines the wedge angle needed for a desired WBLR, this simulation reverses the process by estimating the WBLR that would result from a predefined wedge angle following osteotomy (Fig. 1B) [15]. This simulation reflects the predicted postoperative alignment, assuming that the postoperative intra-articular and soft-tissue conditions correspond to those observed in the preoperative standing, supine, and valgus-stress states, respectively. The simulated Post-WBLRs were designated as simulated Post-WBLR_standing_, Post-WBLR_supine_, and Post-WBLR_valgus_. These simulations allowed for the assessment of which preoperative radiographic condition best matched the actual postoperative standing alignment. Additionally, they evaluated whether planning should depend on a single modality or integrate multiple modalities with appropriate weighting to reduce planning errors.

### Statistical analysis

Patients were stratified into two subgroups on the basis of postoperative alignment: valgus (HKAA ≥ 180°) and varus (HKAA < 180°). This stratification accounts for the distinct intra-articular and soft-tissue conditions associated with each alignment state. Although the surgical goal was to avoid undercorrection, postoperative varus alignment does occur in clinical practice. Therefore, these patients were included to evaluate group-specific trends and to determine whether the optimal planning modality varies under different soft-tissue conditions.

Continuous variables were reported as mean ± standard deviation, while categorical variables were expressed as frequencies and percentages. Welch’s *t*-test or the Mann–Whitney *U* test was used to compare baseline characteristics between the two groups for continuous variables, while the chi-squared test was applied for categorical variables. Differences in radiologic parameters across various preoperative modalities (standing, supine, and valgus-stress) and the postoperative state were assessed using paired *t*-tests. The accuracy of planning for each radiographic modality was evaluated by comparing simulated and actual Post-WBLR, including the mean difference with 95% confidence intervals, paired *t*-test, and root mean square error (RMSE). The correlation and agreement between each simulated Post-WBLR and the actual Post-WBLR were further examined using Pearson correlation coefficients and the ICC (two-way random-effects, absolute agreement), respectively.

To identify independent predictors and derive the regression equation of actual Post-WBLR, linear regression analyses were conducted in the postoperative valgus subgroup, which represents the clinically intended target alignment in MOWHTO. The postoperative varus subgroup was not included in the model because residual undercorrection may reflect a mechanically distinct intra-articular and soft-tissue environment, thus combining these heterogeneous subgroups could have yielded a model poorly tailored to either alignment state. Candidate predictors were initially screened using univariable linear regression (*P* < 0.10), followed by multivariable linear regression with backward elimination (retention criterion *P* < 0.05) to limit potential multicollinearity among predictors. Multicollinearity was further assessed using the variance inflation factor (VIF), and pairwise correlations among actual and simulated Post-WBLR metrics were visualized using a correlation matrix. This process ultimately produced a parsimonious final model for predicting Post-WBLR, retaining only two predictors, thereby minimizing the risk of overfitting. The performance of the resulting regression model was evaluated in terms of both apparent performance and internal validity. As an internal validation step to assess model optimism and overfitting, leave-one-out cross-validation (LOOCV) was performed. To ensure a rigorous validation process and prevent data leakage, the entire variable selection workflow was completely repeated within each LOOCV iteration. To assess the stability of the model, the standard deviation of the regression coefficients was calculated across the LOOCV iterations and compared with the point estimates derived from the entire dataset. The statistical significance of differences in *R*^2^ and ICC between the regression models (both apparent and LOOCV-validated) and the baseline supine-based simulation was evaluated using paired bootstrap resampling with 1000 iterations. Differences in RMSE were evaluated using a paired *t*-test on squared errors. Additionally, the distribution of prediction errors (actual minus predicted Post-WBLR) was visualized using kernel density plots to enable qualitative comparisons of error profiles between models.

To determine the conditions under which single-modality planning may become unreliable—and when a more comprehensive regression-based approach might be necessary—an exploratory receiver operating characteristic (ROC) analysis was performed in the postoperative valgus subgroup. Given that the target WBLR is typically approximately 62.5% [14], with a generally acceptable range of 50–70%, planning outlier was defined as an absolute prediction error exceeding 8%, 9%, and 10% between the actual Post-WBLR and the simulated Post-WBLR based on the most accurate modality. For each threshold, the discriminatory ability of various JLCA-related parameters was assessed using the area under the ROC curve (AUC). The parameter with the highest AUC was chosen as the primary predictor of inaccuracy in single-modality planning, and its optimal cutoff value was determined using Youden’s index.

As variability in follow-up timing may influence postoperative JLCA, and consequently Post-WBLR, a sensitivity analysis was performed restricting the analysis to patients whose postoperative radiographs were obtained within 9–15 months of surgery.

With an overall cohort of 71 patients and 48 patients in the postoperative valgus subgroup (main subgroup), statistical power was adequate for the analyses. In the postoperative valgus subgroup, the study was sufficiently powered to detect small paired differences (minimum detectable *d*_*z*_ = 0.41 for 80% power) at *α* = 0.05. Post hoc power analysis indicated > 99% power to detect the observed correlations. All statistical analyses were conducted using Python (version 3.12.4; Python Software Foundation) along with the scipy, statsmodels, and pingouin libraries. A *P* < 0.05 was considered statistically significant.

## Results

### Baseline characteristics and radiologic parameters

A total of 71 patients were divided into two subgroups on the basis of their 1-year postoperative HKAA: the postoperative valgus group (*n* = 48) and postoperative varus group (*n* = 23). No significant differences were observed between the two subgroups regarding demographic characteristics, including age, sex, body mass index, and preoperative osteoarthritis severity (Table 1).

**Table 1:** Baseline characteristics^a^

Characteristics	Postop valgus subgroup^b^ (*n* = 48)	Postop varus subgroup^b^ (*n* = 23)	*P* value^c^
Age, years	52.4 ± 8.2	47.6 ± 14.0	0.142
Sex			0.565
Male	12 (25.0%)	8 (34.8%)	
Female	36 (75.0%)	15 (65.2%)	
BMI, kg/m^2^	27.8 ± 3.7	25.9 ± 3.8	0.069
Side			0.935
Right	23 (47.9%)	12 (52.2%)	
Left	25 (52.1%)	11 (47.8%)	
K-L grade			0.154
1	7 (14.6%)	0 (0.0%)	
2	17 (35.4%)	10 (43.5%)	
3	24 (50.0%)	13 (56.5%)	
Follow-up timing, months	12.0 (11.0–12.0)	12.0 (11.0–12.0)	0.863

^a^Values are presented as mean ± SD for continuous variables, *n* (%) for categorical variables, and median (interquartile range) for follow-up timing. Postop, postoperative; BMI, body mass index; K-L, Kellgren–Lawrence; HKAA, hip–knee–ankle angle

^b^Postop valgus and varus subgroups were defined by 1-year postoperative HKAA (valgus, HKAA ≥ 180°; varus, HKAA < 180°)

^c^P values were calculated using Welch’s *t*-test for continuous variables and the chi-squared test for categorical variables

Preoperative WBLR and HKAA progressively increased from preoperative standing to supine and further to valgus-stress radiographs (all *P* < 0.001), while JLCA showed a decrease (all *P* < 0.001), in both subgroups (Table 2). Compared with preoperative standing, the 1-year postoperative standing radiographs exhibited significantly greater valgus alignment (all *P* < 0.001 for WBLR and HKAA). Notably, postoperative standing JLCA values fell between the preoperative standing and supine measurements in both the postoperative valgus subgroup (1.8° versus 3.3° and 0.9°) and the postoperative varus subgroup (2.5° versus 4.2° and 1.3°) (Table 2).

**Table 2:** Radiologic parameters measured on preoperative standing, supine, and valgus-stress, and postoperative standing whole-leg radiographs^a^

Radiologic parameters		Preop standing	Preop supine	Preop valgus stress	Postop standing	*P* value^b^	*P* value^c^
Postop valgus subgroup							
WBLR (%)	17.4 ± 10.9		26.7 ± 7.7	44.3 ± 10.2	61.5 ± 7.2^d,e^	< 0.001	< 0.001
HKAA (deg)	173.4 ± 2.3		175.1 ± 1.8	178.9 ± 2.6	182.9 ± 1.6^d,e^	< 0.001	< 0.001
JLCA (deg)	3.3 ± 1.9		0.9 ± 1.7	−1.7 ± 1.7	1.8 ± 1.7^d,e^	< 0.001	< 0.001
MPTA (deg)	84.5 ± 2.4		84.0 ± 2.3	84.1 ± 2.4	92.1 ± 2.2^d,e^	0.077	0.706
∆MPTA_Post–Pre_ (deg)^f^					7.7 ± 2.2		
${l}_{1}/r$ ratio^f^					1.07 ± 0.01		
Back-calculated wedge angle (deg)^f^					8.3 ± 2.3		
Simulated Post-WBLR (%)^f^	52.2 ± 9.5		61.5 ± 8.6	76.7 ± 10.0			
Postop varus subgroup							
WBLR (%)	1.5 ± 14.2		13.9 ± 12.1	34.8 ± 14.0	39.7 ± 10.0^d,e^	< 0.001	< 0.001
HKAA (deg)	170.1 ± 2.9		172.2 ± 2.3	176.7 ± 3.2	178.2 ± 2.1^d,e^	< 0.001	< 0.001
JLCA (deg)	4.2 ± 2.7		1.3 ± 2.1	−1.2 ± 2.2	2.5 ± 2.8^d,e^	< 0.001	< 0.001
MPTA (deg)	83.6 ± 2.2		83.6 ± 2.6	83.8 ± 2.3	90.3 ± 2.2^d,e^	0.883	0.656
∆MPTA_Post–Pre_ (deg)^f^					6.7 ± 2.3		
${l}_{1}/r$ ratio^f^					1.07 ± 0.01		
Back-calculated wedge angle (deg)^f^					7.2 ± 2.5		
Simulated Post-WBLR (%)^f^	33.6 ± 11.7		44.8 ± 8.8	65.5 ± 13.7			

^a^Values are presented as mean ± standard deviation. Full parameters are available in Appendix Table A1. WBLR, weight-bearing line ratio; HKAA, hip–knee–ankle angle; JLCA, joint line convergence angle; MPTA, medial proximal tibial angle; Postop, postoperative; Post, postoperative; Preop, preoperative; Pre, preoperative

^b^Comparison between preoperative standing and preoperative supine radiographs (paired *t*-test)

^c^Comparison between preoperative supine and preoperative valgus-stress radiographs (paired *t*-test)

^d^Significantly different from the value on preoperative standing radiographs (paired *t*-test, *P* < 0.001)

^e^Significantly different from the value on preoperative supine radiographs (paired *t*-test, *P* < 0.008)

^f^Defined and illustrated in Fig. 1

Following the methods illustrated in Fig. 1A, the achieved wedge angle (8.3° ± 2.3° in the postoperative valgus subgroup; 7.2° ± 2.5° in the varus subgroup) was back-calculated using ∆MPTA_Post–Pre_ (7.7° ± 2.2° in the valgus subgroup; 6.7° ± 2.3° in the varus subgroup) and ${l}_{1}/r$ ratio (1.07 ± 0.01 in both subgroups). In the postoperative valgus subgroup, applying the wedge angle to each preoperative radiographic modality (Fig. 1B) resulted in simulated Post-WBLRs of 52.2% ± 9.5%, 61.5% ± 8.6%, and 76.7% ± 10.0% for standing, supine, and valgus-stress radiographs, respectively (Table 2).

### Actual versus simulated postoperative WBLR on each radiographic modality

**Fig. 2:**
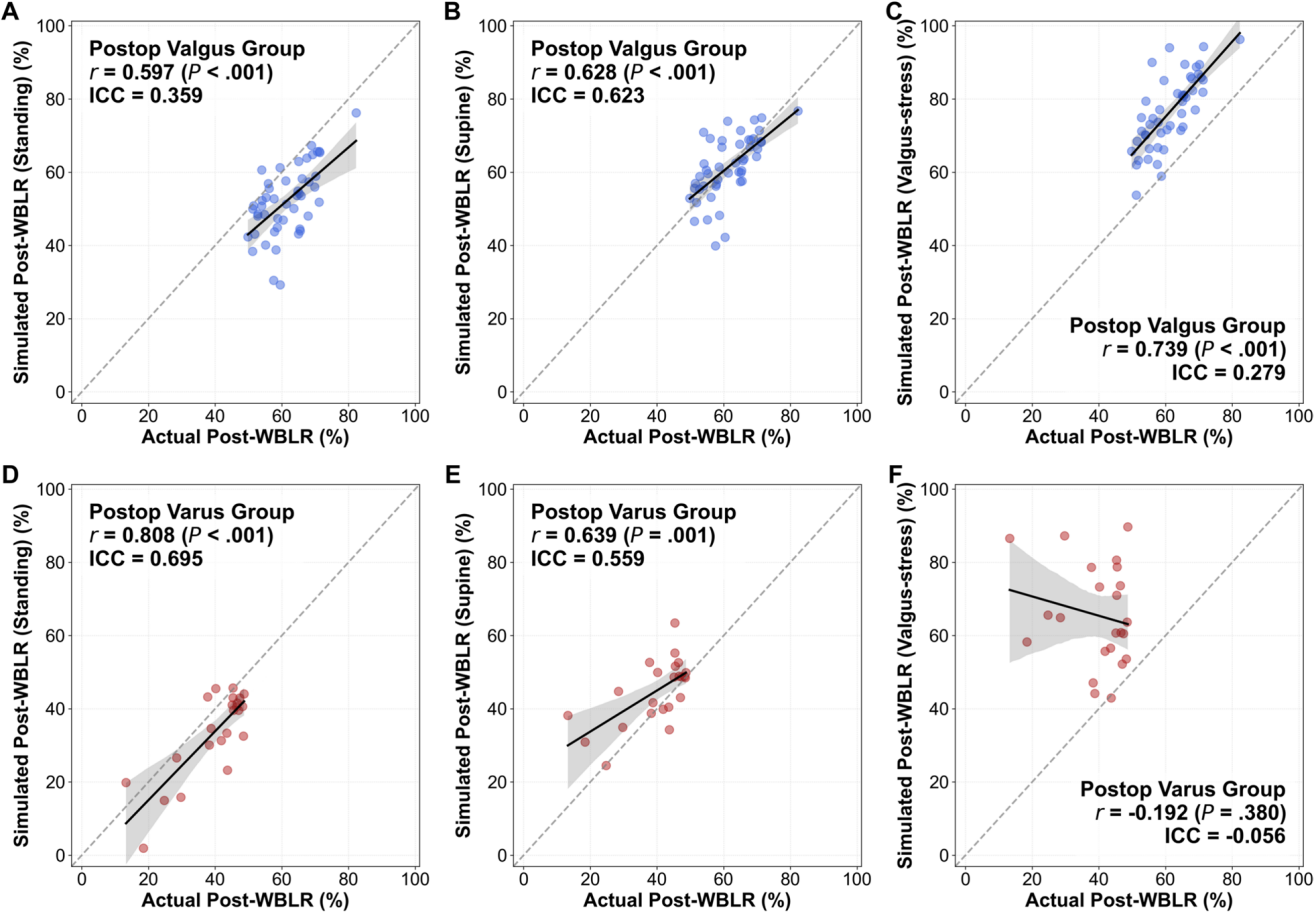
Scatter plots illustrate the correlation and agreement between actual Post-WBLR and simulated Post-WBLR derived from preoperative (**A**, **D**) standing, (**B**, **E**) supine, and (**C**, **F**) valgus-stress whole-leg radiographs. Simulated Post-WBLR was calculated by applying a back-calculated wedge angle to the preoperative radiographs using the Miniaci method. The upper row (**A**–**C**) represents the postoperative valgus subgroup, and the lower row (**D**–**F**) represents the postoperative varus subgroup. Dashed gray lines indicate the line of identity (*y* = *x*; perfect planning). Solid black lines depict linear regression fits, with shaded areas representing 95% confidence intervals. Pearson’s correlation coefficients (*r*), corresponding *P* values, and ICCs are displayed within each panel. Post, postoperative; Postop, postoperative; Post-WBLR, postoperative weight-bearing line ratio; ICC, intraclass correlation coefficient

In the postoperative valgus subgroup, the supine-based simulation most closely approximated the actual Post-WBLR (Table 3, Fig. 2A–C). The simulated Post-WBLR_supine_ showed no significant systemic bias relative to the actual Post-WBLR (mean difference, 0.0% ± 6.9%; *P* = 0.983) and exhibited the lowest error (RMSE = 6.8; *P* < 0.001 for both standing and valgus-stress). In contrast, the standing-based simulation significantly underestimated the actual Post-WBLR (mean difference, −9.3% ± 7.8%; *P* < 0.001) with a higher RMSE (12.1), whereas valgus-stress simulation significantly overestimated it (mean difference, 15.2% ± 6.7%; *P* < 0.001) with the largest RMSE (16.6) and poor agreement (ICC = 0.279). Despite this systemic overestimation and poor agreement, the valgus-stress simulation showed strong correlation with the actual Post-WBLR (*r* = 0.739, *P* < 0.001).

**Table 3:** Comparison of actual Post-WBLR and simulated Post-WBLR (accuracy of preoperative planning) derived from standing, supine, and valgus-stress whole-leg radiographs^a^

	Postop valgus subgroup				Postop varus subgroup			
	Mean difference^c^	*P* value^d^	RMSE	*P* value^e^	Mean difference^c^	*P* value^d^	RMSE	*P* value^e^
Simulated Post-WBLR_standing_^b^	−9.3 ± 7.8 [−11.6, −7.0]	< 0.001	12.1	< 0.001	−6.1 ± 6.9 [−9.1, −3.1]	< 0.001	9.1	0.393
Simulated Post-WBLR_supine_^b^	0.0 ± 6.9 [−2.0, 2.0]	0.983	6.8	–	5.1 ± 8.1 [1.6, 8.6]	0.006	9.4	–
Simulated Post-WBLR_valgus_^b^	15.2 ± 6.7 [13.2, 17.2]	< 0.001	16.6	< 0.001	25.8 ± 18.5 [17.8, 33.8]	< 0.001	31.5	< 0.001

^a^Post-WBLR, postoperative weight-bearing line ratio; RMSE, root mean square error; Postop, postoperative; CI, confidence interval

^b^Defined and illustrated in Fig. 1

^c^Mean ± standard deviation [95% CI]

^d^Comparison between the simulated and the actual Post-WBLR (paired *t*-test)

^e^Comparison of RMSE versus the supine-based simulation (Wilcoxon signed-rank test on squared errors)

In the postoperative varus subgroup, standing-based simulation yielded a significantly smaller WBLR (simulated Post-WBLR_standing_) than the actual postoperative value (mean difference, −6.1% ± 6.9%; *P* < 0.001), whereas supine-based simulation produced a significantly larger WBLR than the actual postoperative value (mean difference, 5.1% ± 8.1%; *P* = 0.006) (Table 3, Fig. 2D–F). However, the magnitude of error was similar between standing and supine simulations (RMSE, 9.1 versus 9.4; *P* = 0.393). Valgus-stress simulation significantly overestimated postoperative alignment (mean difference, 25.8% ± 18.5%; *P* < 0.001), exhibiting a considerably larger error (RMSE = 31.5; *P* < 0.001 versus supine). These distribution patterns are illustrated in scatter plots in Fig. 2. Similar findings were observed when the analysis was restricted to patients with a follow-up of 9–15 months (sensitivity analysis; *n* = 57 [80.3% of the total cohort]; Appendix Table A2).

### Supine-based simulation versus regression model-based simulation

**Fig. 3:**
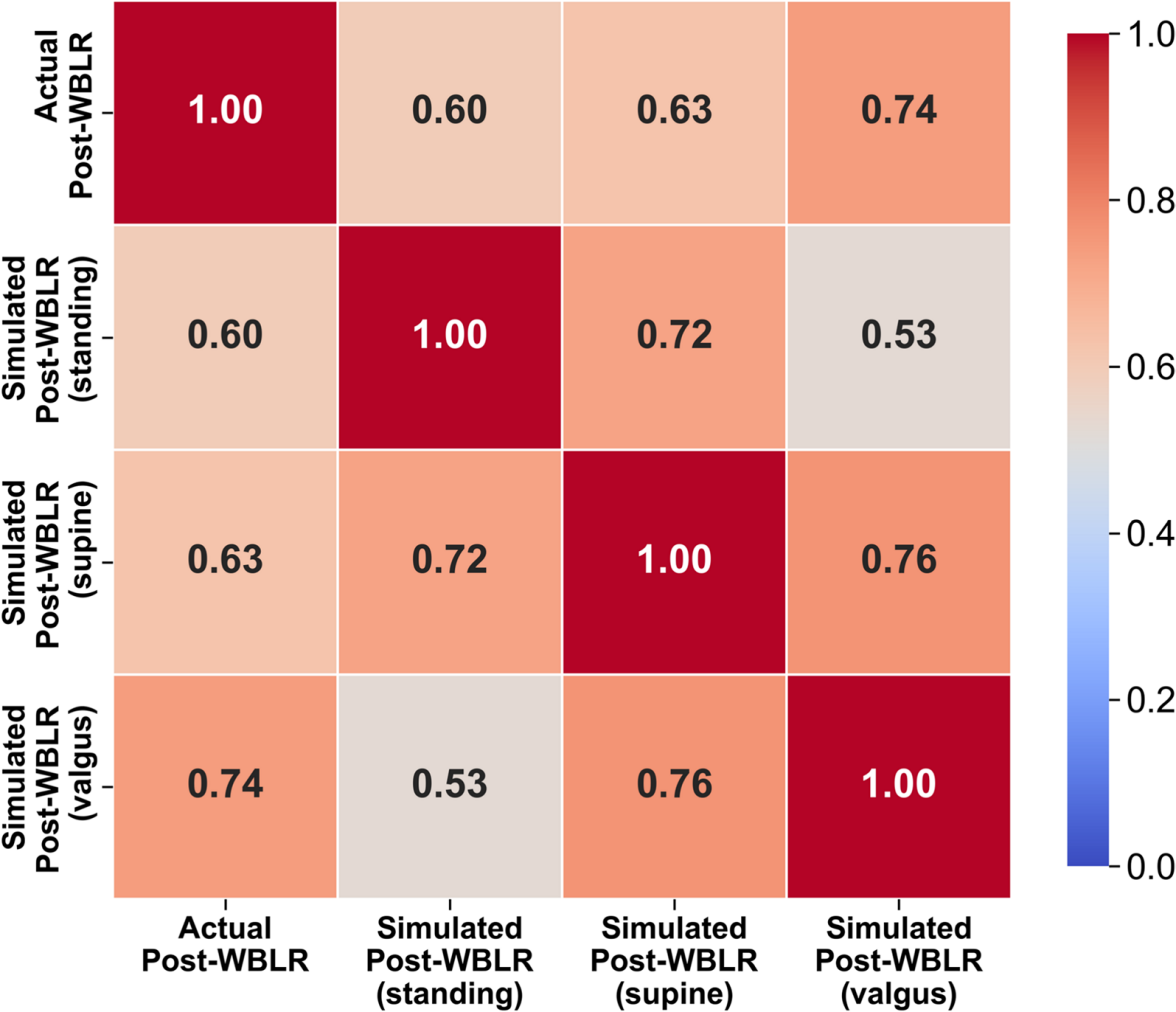
Correlation matrix (heat map) displaying Pearson’s correlation coefficients (*r*) between the actual postoperative WBLR and simulated values derived from standing, supine, and valgus-stress radiographs in the postoperative valgus group. Post, postoperative; WBLR, weight-bearing line ratio

In the postoperative valgus subgroup, univariable linear regression identified several factors that met the screening criterion of *P* < 0.10 for predicting actual Post-WBLR: sex, preoperative valgus-stress WBLR, back-calculated wedge angle, simulated Post-WBLR_standing_, simulated Post-WBLR_supine_, and simulated Post-WBLR_valgus_ (Appendix Table A3). The subsequent multivariable analysis produced a predictive regression model (*R*^2^ = 0.606, adjusted *R*^2^ = 0.589, *P* < 0.001), which included simulated Post-WBLR_standing_ and simulated Post-WBLR_valgus_ as independent predictors (Table 4). The multicollinearity between these predictors was low (VIF = 1.381), and they showed the lowest mutual correlation (*r* = 0.53) among the modality combinations (Table 4, Fig. 3). When the analysis was restricted to patients with a follow-up of 9–15 months (sensitivity analysis (*n* = 38 [79.2%] in the postoperative valgus subgroup), the regression model yielded comparable performance (*R*^2^ = 0.541, adjusted *R*^2^ = 0.515, *P* < 0.001, RMSE = 4.4%).

**Table 4:** Multivariable linear regression model for predicting Post-WBLR in the postoperative valgus subgroup (*R*^2^ = 0.606, adjusted *R*^2^ = 0.589, *P* < 0.001)^a^

Dependent variable	Explicative variables	Unstandardized coefficient			Standardized coefficient^b^		VIF
		*B*	SE (*B*)	95% CI	β	*P* value	
Actual Post-WBLR	Constant	17.66	5.33	6.92–28.40		0.002	
	Simulated Post-WBLR_standing_	0.218	0.083	0.05–0.39	0.288	0.012	1.381
	Simulated Post-WBLR_valgus_	0.423	0.079	0.26–0.58	0.588	< 0.001	1.381

Regression model for Post-WBLR = 17.7 + 0.218 × Simulated Post-WBLR_standing_ + 0.423 × Simulated Post-WBLR_valgus_

^a^Variables with *P* < 0.10 in univariable analysis (Appendix Table A3) were initially included in the multivariable model. Multivariable linear regression analysis was performed using a backward elimination approach with a significance level of ≥ 0.05 for removal. Post-WBLR, postoperative weight-bearing line ratio; VIF, variance inflation factor; SE, standard error; CI, confidence interval

^b^Standardized coefficients were calculated to allow comparison of the relative contribution of each variable

**Fig. 4:**
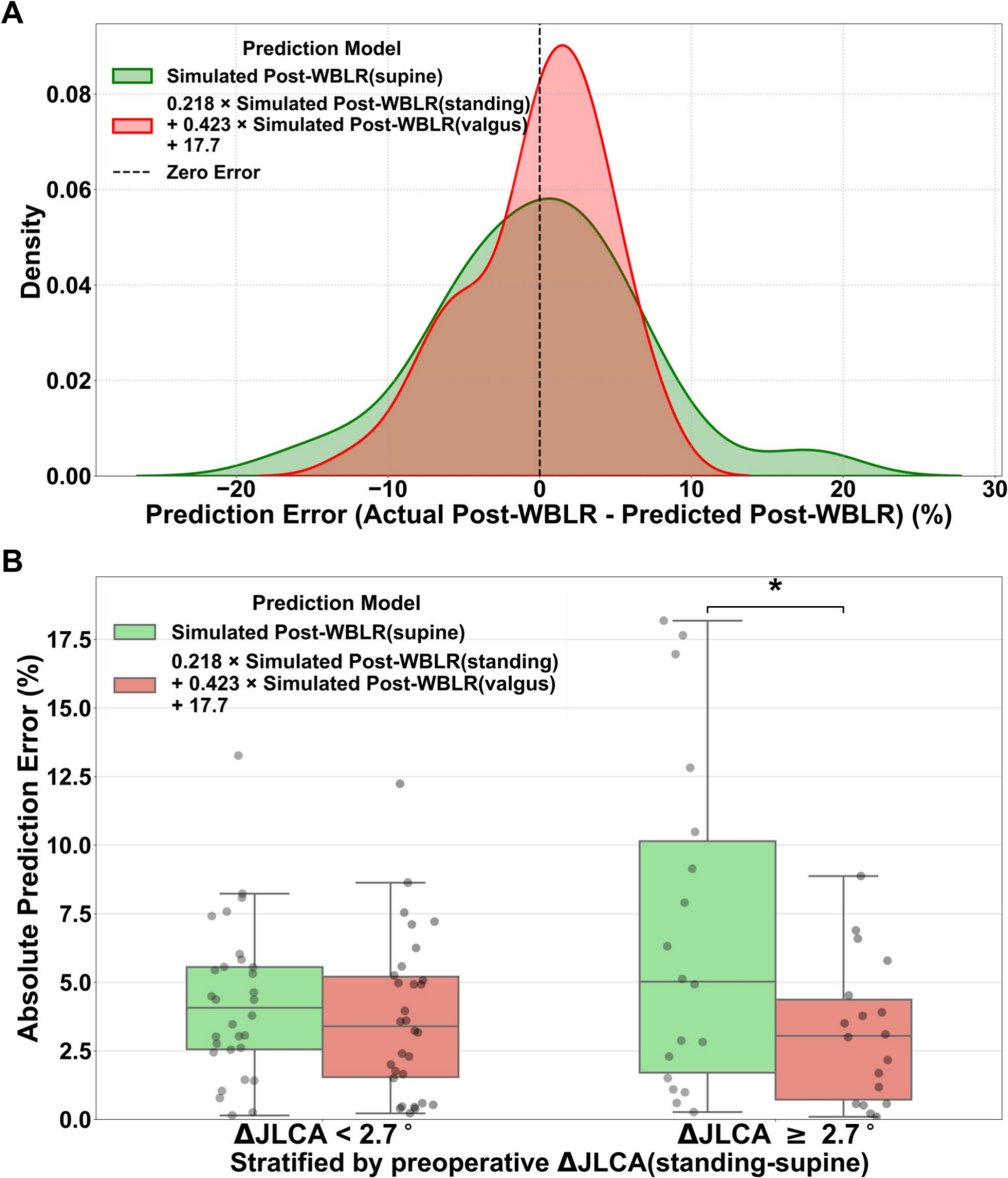
Comparison of prediction errors between supine simulation (green) and a regression model that incorporates standing and valgus-stress simulations (red) in the postoperative valgus subgroup. Prediction errors were defined as the actual values minus the predicted Post-WBLR. **A** Kernel density plots display the distribution of prediction errors for each model. The dashed line indicates perfect prediction. **B** Box plots of absolute prediction errors stratified by preoperative ∆JLCA_standing–supine_ (< 2.7° versus ≥ 2.7°). Boxes indicate the interquartile range with median lines. An asterisk denotes a statistically significant between-model difference (Wilcoxon signed-rank test). Post-WBLR, postoperative weight-bearing line ratio; JLCA, joint line convergence angle

Compared with the supine-based simulation, the multimodal regression model demonstrated superior predictive performance across all metrics: *R*^2^ (0.606 versus 0.080, *P* < 0.001), ICC (0.759 versus 0.623, *P* = 0.022), and RMSE (4.5 versus 6.8, *P* = 0.013) (Table 5). LOOCV supported the stability and internal validity of the regression model, showing only a marginal decrease in predictive performance (LOOCV–*R*^2^ = 0.551 and LOOCV–RMSE = 4.8). The regression coefficients remained stable across LOOCV iterations, with standard deviations of 0.011 (5.1% of the point estimate) for the Simulated Post-WBLR_standing_ coefficient and 0.012 (2.9% of the point estimate) for the Simulated Post-WBLR_valgus_ coefficient. Under this strict internal cross-validation, the regression model maintained statistically significant superiority over the supine-based simulation (*P* < 0.001 for *R*^2^ difference; *P* = 0.024 for RMSE difference). Kernel density plots further illustrated that relying solely on the supine modality led to a higher frequency of large deviations from the actual Post-WBLR, while the regression approach reduced extreme outliers (Fig. 4A).

**Table 5:** Predictive performance and internal validation of the multimodal regression model compared with supine-based simulation for predicting actual Post-WBLR^a^

Prediction model (postoperative valgus subgroup)	*R*^2^	*P* value^b^	ICC	*P* value^c^	RMSE	*P* value^d^
Regression model for Post-WBLR^e^ (apparent performance)	0.606	< 0.001	0.759	0.022	4.5	0.013
Regression model for Post-WBLR^e^ (LOOCV internal validation)	0.551	< 0.001	–	–	4.8	0.024
Simulated Post-WBLR_supine_	0.080	–	0.623	–	6.8	–

^a^Post-WBLR, postoperative weight-bearing line ratio; ICC, intraclass correlation coefficient; RMSE, root mean square error; LOOCV, leave-one-out cross-validation

^b^Comparison of *R*^2^ versus the supine-based simulation (bootstrapping, 1000 iterations)

^c^Comparison of ICC versus the supine-based simulation (bootstrapping, 1000 iterations)

^d^Comparison of RMSE versus the supine-based simulation (paired *t*-test on squared errors)

^e^Regression model for Post-WBLR = 17.7 + 0.218 × simulated Post-WBLR_standing_ + 0.423 × simulated Post-WBLR_valgus_

### Application in preoperative planning

**Fig. 5:**
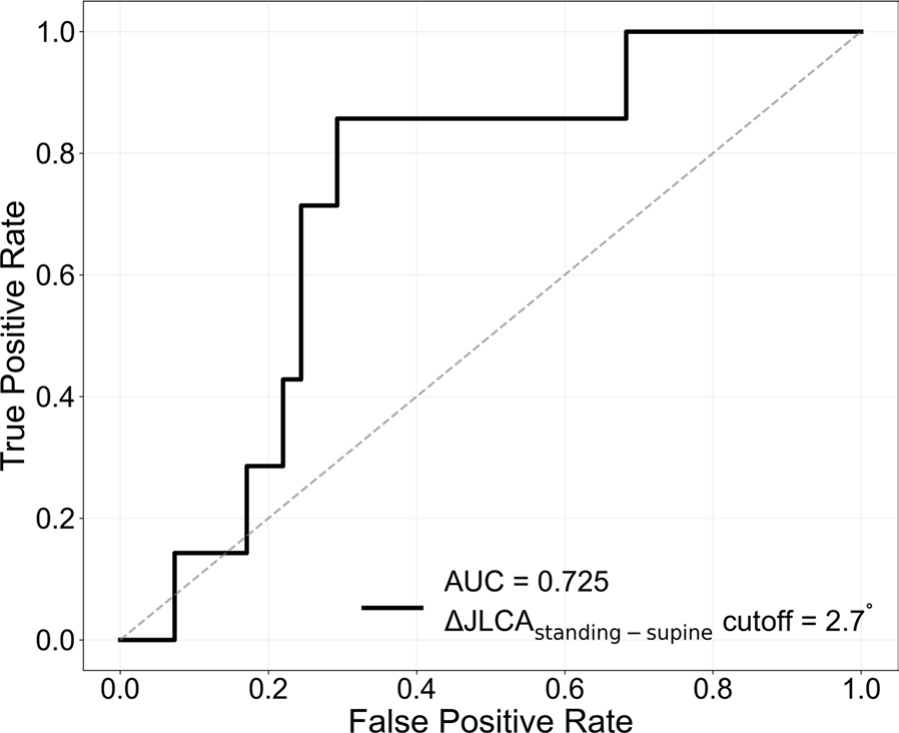
Receiver operating characteristic curve assessing the difference in JLCA between standing and supine radiographs (ΔJLCAstanding–supine) as a predictor of high prediction error in supine-based planning. AUC, area under the receiver operating characteristic curve; JLCA, joint line convergence angle; WBLR, weight-bearing line ratio

Although the incorporation of standing and valgus-stress simulations through the regression model improved accuracy and reduced outliers, supine-based planning alone already demonstrated acceptable average performance. Therefore, the routine application of multi-modality simulation and regression calculations may be unnecessarily complex. Accordingly, an ROC analysis was performed to identify the conditions under which supine-only planning becomes unreliable. At the 9% prediction error threshold, preoperative ∆JLCA_standing–supine_ showed the highest discriminatory ability for predicting planning outlier (AUC = 0.725; Appendix Table A4), with an optimal cutoff value of 2.7° (Fig. 5).

In patients with preoperative ∆JLCA_standing–supine_ < 2.7°, there were no significant differences in absolute prediction errors between the regression model and the supine-based simulation (3.7% ± 2.9% versus 4.3% ± 2.8%; *P* = 0.382; Fig. 4B). However, when ∆JLCA_standing–supine_ ≥ 2.7°, the regression model significantly reduced prediction error compared with the supine-based simulation (3.2% ± 2.6% versus 6.8% ± 6.1%; *P* = 0.014) and showed a markedly lower RMSE (4.0% versus 9.0%; *P* < 0.001).

**Fig. 6:**
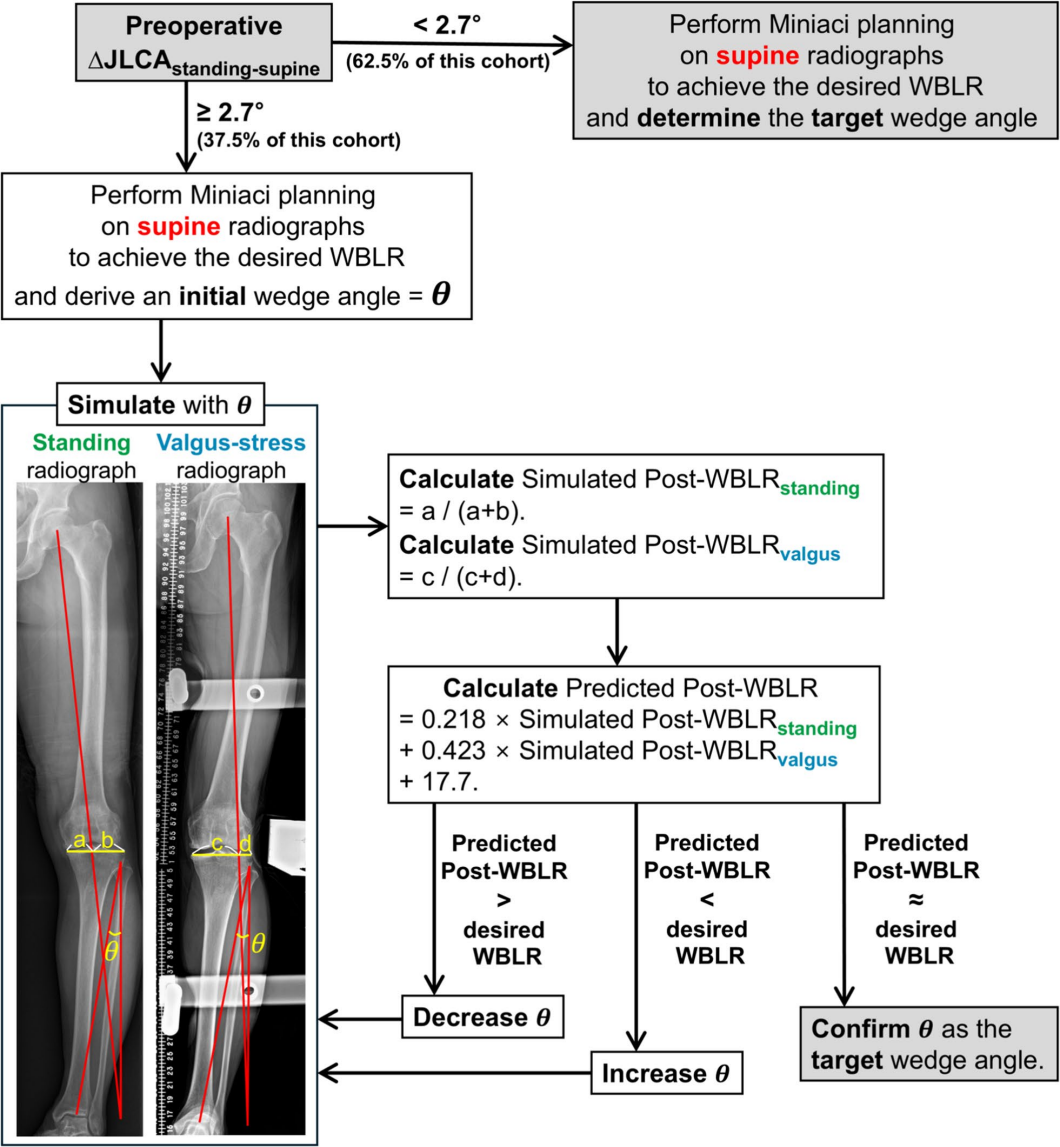
Application of the results to preoperative planning. The proposed flowchart illustrates an exploratory stepwise approach for determining the target wedge angle in medial open-wedge high tibial osteotomy. The planning strategy is determined based on the preoperative difference in JLCA between standing and supine radiographs (∆JLCA_standing–supine_). The percentages indicate the patient distribution within this cohort according to the 2.7° threshold. Gray-shaded boxes indicate the starting and ending points of the workflow. For patients with ∆JLCA_standing–supine_ < 2.7°, conventional Miniaci planning on supine radiographs may be sufficient. For patients with ∆JLCA_standing–supine_ ≥ 2.7°, the regression model-based simulation may be considered. An initial wedge angle (*θ*) is derived from supine planning and virtually applied to standing and valgus-stress radiographs to calculate the predicted Post-WBLR using the regression formula. The wedge angle is iteratively adjusted and resimulated until the predicted WBLR approximates the desired target WBLR. JLCA, joint line convergence angle; WBLR, weight-bearing line ratio; Post, postoperative

On the basis of these findings, an exploratory stepwise workflow for preoperative planning is proposed (Fig. 6). If ∆JLCA_standing–supine_ < 2.7°, conventional Miniaci planning on supine radiographs appears sufficient owing to its simplicity and low error rate. However, when the value is ≥ 2.7°, the regression model may be considered to mitigate the risk of planning outliers. In this case, the wedge angle initially derived from the supine planning should be reapplied to standing and valgus-stress radiographs to obtain simulated Post-WBLR values. These values are then entered into the regression equation to estimate the predicted postoperative alignment. If the predicted Post-WBLR deviates from the desired target, the wedge angle should be adjusted iteratively until the predicted value aligns with the surgical goal.

## Discussion

The main finding of this study was that effective preoperative planning for MOWHTO requires the consideration of whole-leg radiographs obtained in standing, supine, and valgus-stress positions to improve postoperative alignment prediction and reduce the risks of under- or overcorrection. In the postoperative valgus subgroup, which reflects the desired alignment, simulation on supine radiographs showed the least systemic bias among the three modalities, with virtually no mean difference from the actual postoperative alignment. Combining simulations on standing and valgus-stress radiographs through a regression model further improved predictive performance and reduced planning outliers compared with the supine-only simulation. These results suggest an exploratory adaptive planning approach (Fig. 6): given the complexity of routine multimodal regression calculations, supine radiographs can serve as a practical default modality for planning, whereas multimodal integration may be beneficial when intra-articular alignment is highly load-dependent—particularly when ∆JLCA_standing–supine_ ≥ 2.7°.

Prior studies have consistently demonstrated that supine radiographs provide a more accurate basis for MOWHTO planning than standing radiographs [11, 14, 15 ], and Sakai et al. [14] have recently utilized similar ROC cutoff analyses to identify patients at risk of planning error. The present study builds upon this foundation by investigating three novel aspects to enhance predictive precision: (1) the first clinical application of valgus-stress whole-leg radiographs for preoperative simulation, (2) the derivation of a multimodal regression model and planning workflow integrating three radiographic modalities, and (3) a mathematically rigorous back-calculation of the wedge angle from ∆MPTA_Post–Pre_ using the ${l}_{1}/r$ ratio.

Because MOWHTO transitions a varus limb into a valgus state, traditional standing and supine radiographs [10–16] may not fully replicate the postoperative biomechanical environment, where weight-bearing induces valgus-producing torque and tensions the medial ligaments. Although valgus-stress radiographs may be biomechanically suited to simulate the postoperative soft tissue environment, previous research [4, 17, 20 –] has focused solely on valgus-stress localized knee radiographs for JLCA analysis, rather than utilizing whole-leg radiographs for comprehensive planning or simulations. By simulating on a continuous spectrum from varus standing to neutral supine and valgus-stress positions, we identified where the postoperative intra-articular alignment resides and determined how to optimally combine the information from each modality to predict Post-WBLR.

Methodologically, a mathematical approach was introduced to accurately back-calculate the achieved opening wedge angle on the basis of postoperative radiographic parameters. Several previous studies merely added or subtracted angular parameters such as wedge angle, MPTA, and JLCA without considering their different centers of rotation and often assumed that the wedge angle was identical to ∆MPTA_Post–Pre_ [3, 15, 24,23 ]. However, in the present study, the back-calculated wedge angle was approximately 1.07 times ∆MPTA_Post–Pre_, corresponding to a difference of about 0.5°. Since proposed clinical adjustments to improve planning accuracy in previous studies [3, 4, 25 ] are often as fine as 1°, this 0.5° discrepancy is not negligible. Thus, incorporating the ${l}_{1}/r$ ratio when deriving the wedge angle provided meaningful refinement. This approach enabled the analysis to focus on planning error while minimizing noise arising from surgical execution error.

Consistent with previous studies [11, 15, 23 ], postoperative standing JLCA values were found to be between the preoperative standing and supine measurements in both subgroups (Table 2). These findings suggest that postoperative intra-articular alignment typically falls between the standing and supine states, regardless of whether the sMCL is released via transection [15] or merely elevated subperiosteally as in our study. Even in the postoperative valgus subgroup, JLCA was found to be between preoperative standing and supine values, rather than between the supine and valgus-stress values. This phenomenon can be attributed to how load is distributed across the knee joint during standing. Since the body’s center of mass is located between the two limbs, approximately 70% of the vertical load is transmitted through the medial compartment even in neutral alignment [26]. As a result, medial compartment loading remains greater than lateral loading, even after correction to a slight valgus alignment.

Interestingly, although the postoperative intra-articular alignment, represented by JLCA, fell between the preoperative standing and supine conditions, the overall mechanical alignment, represented by WBLR, was most accurately predicted using supine-based planning (Table 3, postoperative valgus subgroup). This may be due to the slight lengthening of the tibia postoperatively [2], which shifts the ankle laterally and thus increases the WBLR. While planning based on standing radiographs remains common in many institutions [5, 19, 24 ], our finding that supine planning is superior to standing planning aligns with Shin et al. [15]. This suggests that the reduced knee adduction moment after MOWHTO creates a mechanical environment that is functionally similar to the preoperative non-weight-bearing (supine) state [15, 27 ]. Our subgroup analysis further revealed that, in residual postoperative varus cases, the actual Post-WBLR lay between the standing- and supine-based simulated values, indicating that this undercorrected subgroup reflected an intermediate mechanical state. Regardless of postoperative alignment, standing-based planning systematically underestimated Post-WBLR, while valgus-stress planning tended to overestimate postoperative alignment, reflecting modality-dependent differences in preoperative JLCA (Table 3, Fig. 2).

Despite the systemic overestimation with the highest RMSE and poor agreement, valgus-stress simulations demonstrated a strong correlation with actual Post-WBLR in the postoperative valgus subgroup (Fig. 2C). This pattern suggests that valgus-stress radiographs do not provide accurate stand-alone alignment estimates but may capture interindividual variability in correctable intra-articular alignment, including the maximal extent of medial joint opening. This observation is consistent with previous research indicating that JLCA or articular gap measurements from valgus-stress radiographs affect correction accuracy and postoperative JLCA changes [4, 17, 20 –]. Notably, in the multivariable regression model, the simulated WBLR derived from valgus-stress radiographs emerged as the most significant predictor of Post-WBLR, exhibiting the largest coefficient.

The regression model also identified simulated Post-WBLR_standing_ as an independent predictor. The two predictors exhibited low multicollinearity and a modest correlation, suggesting that they provide complementary radiologic information. Standing radiographs capture baseline intra-articular alignment under physiological loading and reflect the fixed component of deformity that may lead to undercorrection. In contrast, valgus-stress radiographs illustrate the correctable component and capture the potential limit of overcorrection. These components are influenced by a complex interplay of factors, including periarticular soft-tissue laxity, lateral joint lift-off, meniscus extrusion, and cartilage wear [18, 19, 29,24,28 ]. The regression model effectively estimates postoperative alignment between these two extremes of undercorrection and overcorrection. While supine-only planning is generally accurate, the regression model showed superior *R*^2^, ICC, and RMSE values, and its performance remained stable and internally valid after LOOCV with only marginal shrinkage in *R*^2^ and RMSE (Table 5). Furthermore, the high stability of the regression coefficients across LOOCV iterations confirms that the model is not overfitted to individual data points. This supports the added predictive value of incorporating the dynamic information from standing and valgus-stress radiographs, which capture the fixed and correctable components, respectively. As shown in Fig. 4A, the model reduced both undercorrection and overcorrection compared with supine-only planning.

However, routine multimodal planning and regression calculations can be clinically cumbersome. Therefore, identifying patients in whom supine-based planning is more likely to yield large prediction errors may improve practical usability. In this study, ∆JLCA_standing–supine_ ≥ 2.7° showed the best discriminatory performance for larger errors in supine-based planning, although the moderate AUC (0.725) supports its use as an exploratory decision-support marker rather than a definitive clinical cutoff. Previous studies have also reported the significance of ∆JLCA_standing–supine_ as an indicator of planning outlier [12, 16 ]. The present study expands on these findings by demonstrating that planning outliers can be mitigated through simulation and regression-based prediction (Fig. 6). A large reduction in JLCA from standing to supine indicates that the medial soft tissues are not severely constrained by contracture [30] or by pseudocontracture caused by underlying osteophytes [31]. In such cases, predicting the degree of postoperative medial ligament stretch based solely on supine radiographs is challenging. Valgus-stress radiographs provide unique information regarding the physiological limits of medial soft-tissue elongation. Incorporating this information into the planning process enhanced prediction accuracy.

The predictive value of valgus-stress radiographs—and consequently, the proposed regression model—is substantially limited in the postoperative varus subgroup. This limitation fundamentally stems from the underlying philosophy of our model, which relies on replicating the intended postoperative biomechanical environment in preoperative radiographs. Because the maximal medial limit captured by valgus-stress imaging is only realized when the joint transitions into valgus, its application in residual varus cases leads to extreme overestimation (mean difference, 25.8% ± 18.5%; Table 3) with no correlation to actual outcomes (*r* = −0.192, ICC = −0.056; Fig. 2F). Although a universally applicable “optimal correction” target may not exist, slight valgus remains a widely accepted clinical goal [32, 33 ]. When aiming for this general target, clinicians can utilize the proposed planning flowchart, which flexibly accommodates a surgeon-defined target WBLR (Fig. 6). Conversely, for patients at a high risk of residual varus—such as those with severe preoperative deformity or soft-tissue contracture—valgus-stress imaging provides no added predictive value. In these cases, clinicians may consider defaulting to supine-based planning, which exhibited the lowest systemic bias independent of postoperative alignment (Table 3).

This study has several limitations. First, while back-calculating the wedge angle from ∆MPTA was designed to isolate planning error by assuming negligible surgical error, the calculated wedge angle may still differ from the actual intraoperative wedge angle. Specifically, variability in identifying the lateral hinge point, projection artifacts, and the inherent limitations of two-dimensional radiographs for representing three-dimensional joint deformity may have affected the calculated wedge angle. As a result, the present method cannot completely isolate planning error, which ultimately influences the simulation results. Second, this study was a single-center retrospective study, which carries the inherent limitations. LOOCV assesses only internal stability and cannot substitute for external validation. Prospective and external validation is necessary to confirm the clinical utility of the proposed planning workflow. Third, the main regression model was derived from the postoperative valgus subgroup, which was defined by the postoperative outcome. Although this choice was intended to avoid combining mechanically heterogeneous subgroups and to model the clinically desired valgus state, it limits the interpretability and generalizability of the model in unselected populations. Fourth, the sMCL was elevated subperiosteally without transection to maintain the option for future knee arthroplasty. Although Sato et al. demonstrated that even complete sMCL release does not cause permanent postoperative valgus instability at 1 year [34], the altered ΔJLCA pattern associated with routine sMCL transection may affect the regression coefficients—particularly the 0.423 coefficient for valgus-stress simulations—and the optimal ΔJLCA_standing–supine_ cutoff of 2.7°, potentially requiring recalibration of the proposed model in such settings. Fifth, all radiographic alignment measurements were performed using a validated AI-assisted software system (CONNECTEVE; previously published ICC, 0.936–0.997 [21]). However, intra- and interobserver reliability for the specific measurements in this cohort were not separately validated, which represents a limitation. Finally, this study concentrated on radiologic parameters rather than surgical decision-making or patient-reported outcomes, and additional research is needed to determine whether the proposed exploratory workflow improves real-world alignment accuracy or clinical outcomes.

## Conclusions

In this retrospective single-center cohort, supine radiographs provided the most accurate single-modality basis for preoperative planning in MOWHTO. In the postoperative valgus group, standing and valgus-stress radiographs appeared to provide complementary information on fixed and correctable components of intra-articular alignment, respectively. Integrating these modalities through a regression model improved prediction accuracy and reduced planning outliers. While further validation is required for routine clinical use, a stepwise planning workflow—using supine radiographs as the default modality and multimodal integration when ∆JLCA_standing–supine_ ≥ 2.7°—may serve as an exploratory framework for reducing the risk of planning-related undercorrection or overcorrection.

## Supplementary Information

Additional file 1.

## Data Availability

The datasets analyzed during the current study are not publicly available owing to institutional restrictions on data privacy but are available from the corresponding author on reasonable request.

## Abbreviations

aHKAA: Arithmetic hip–knee–ankle angle
AUC: Area under the receiver operating characteristic curve
BMI: Body mass index
CI: Confidence interval
HKAA: Hip–knee–ankle angle
ICC: Intraclass correlation coefficient
JLCA: Joint line convergence angle
JLOA: Joint line obliquity angle
K-L: Kellgren–Lawrence
LOOCV: Leave-one-out cross-validation
mLDFA: Mechanical lateral distal femoral angle
MOWHTO: Medial open-wedge high tibial osteotomy
MPTA: Medial proximal tibial angle
Post-WBLR: Postoperative weight-bearing line ratio
RMSE: Root mean square error
ROC: Receiver operating characteristic
SD: Standard deviation
SE: Standard error
sMCL: Superficial medial collateral ligament
VIF: Variance inflation factor
WBLR: Weight-bearing line ratio
