# Supplementary material for: Complementary roles of standing, supine, and valgus-stress whole-leg radiographs in medial open-wedge high tibial osteotomy planning: supine minimizes bias, whereas standing and valgus-stress reduce outliers
Source: Knee Surg Relat Res. 2026 Aug 6;38:38. doi: 10.1186/s43019-026-00340-6 (PMC13450190; doi:10.1186/s43019-026-00340-6)
Supplement: Supplementary file 1 [file 43019_2026_340_MOESM1_ESM.docx]

**Supplementary material**

**Appendix Table A1.** Radiologic parameters measured on preoperative standing, supine, and valgus-stress, and postoperative standing whole-leg radiographs^a^

| Radiologic Parameters | | Preop Standing | Preop Supine | Preop  Valgus-stress | Postop Standing | *P* value^b^ | *P* value^c^ | *P* value^d^ |
| --- | --- | --- | --- | --- | --- | --- | --- | --- |
| Postop Valgus Group | |  |  |  |  |  |  |  |
| WBLR (%) | 17.4 ± 10.9 | | 26.7 ± 7.7 | 44.3 ± 10.2 | 61.5 ± 7.2 | <.001 | <.001 | <.001 |
| HKAA (deg) | 173.4 ± 2.3 | | 175.1 ± 1.8 | 178.9 ± 2.6 | 182.9 ± 1.6 | <.001 | <.001 | <.001 |
| mLDFA (deg) | 87.8 ± 1.7 | | 87.8 ± 1.6 | 87.5 ± 1.6 | 87.3 ± 2.0 | .915 | .060 | .131 |
| JLCA (deg) | 3.3 ± 1.9 | | 0.9 ± 1.7 | -1.7 ± 1.7 | 1.8 ± 1.7 | <.001 | <.001 | <.001 |
| JLOA (deg) | -3.2 ± 2.3 | |  |  | 0.0 ± 2.3 |  |  | <.001 |
| MPTA (deg) | 84.5 ± 2.4 | | 84.0 ± 2.3 | 84.1 ± 2.4 | 92.1 ± 2.2 | .077 | .706 | <.001 |
| ∆MPTA_Post-Pre_ (deg)^e^ |  | |  |  | 7.7 ± 2.2 |  |  |  |
| $l_{1}/r$ ratio^e^ |  | |  |  | 1.07 ± 0.01 |  |  |  |
| Back-calculated  wedge angle (deg)^e^ |  | |  |  | 8.3 ± 2.3 |  |  |  |
| Simulated  Post-WBLR (%)^f^ | 52.2 ± 9.5 | | 61.5 ± 8.6 | 76.7 ± 10.0 |  |  |  |  |
| Postop Varus Group |  | |  |  |  |  |  |  |
| WBLR (%) | 1.5 ± 14.2 | | 13.9 ± 12.1 | 34.8 ± 14.0 | 39.7 ± 10.0 | <.001 | <.001 | <.001 |
| HKAA (deg) | 170.1 ± 2.9 | | 172.2 ± 2.3 | 176.7 ± 3.2 | 178.2 ± 2.1 | <.001 | <.001 | <.001 |
| mLDFA (deg) | 89.2 ± 2.5 | | 89.6 ± 2.6 | 89.0 ± 2.3 | 89.5 ± 3.2 | .170 | .062 | .301 |
| JLCA (deg) | 4.2 ± 2.7 | | 1.3 ± 2.1 | -1.2 ± 2.2 | 2.5 ± 2.8 | <.001 | <.001 | <.001 |
| JLOA (deg) | -2.3 ± 1.8 | |  |  | 0.7 ± 2.8 |  |  | <.001 |
| MPTA (deg) | 83.6 ± 2.2 | | 83.6 ± 2.6 | 83.8 ± 2.3 | 90.3 ± 2.2 | .883 | .656 | <.001 |
| ∆MPTA_Post-Pre_ (deg)^e^ |  | |  |  | 6.7 ± 2.3 |  |  |  |
| $l_{1}/r$ ratio^e^ |  | |  |  | 1.07 ± 0.01 |  |  |  |
| Back-calculated  wedge angle (deg)^e^ |  | |  |  | 7.2 ± 2.5 |  |  |  |
| Simulated  Post-WBLR (%)^f^ | 33.6 ± 11.7 | | 44.8 ± 8.8 | 65.5 ± 13.7 |  |  |  |  |

^a^Values are presented as mean ± standard deviation. WBLR, weight-bearing line ratio; HKAA, hip-knee-ankle angle; mLDFA, mechanical lateral distal femoral angle; JLCA, joint line convergence angle; JLOA, joint line obliquity angle; MPTA, medial proximal tibial angle; Postop, postoperative; Post, postoperative; Preop, preoperative; Pre, preoperative.

^b^Comparison between preoperative standing and preoperative supine radiographs (paired t-test).

^c^Comparison between preoperative supine and preoperative valgus-stress radiographs (paired t-test).

^d^Comparison between preoperative and 1-year postoperative standing radiographs (paired t-test).

^e^Defined and illustrated in Figure 1. ∆MPTA_Post-Pre_ = Postop standing MPTA – Preop standing MPTA.

^f^Simulated Post-WBLR was obtained using the Miniaci method by applying the back-calculated wedge angle (derived from ∆MPTA_Post-Pre_) to each preoperative radiograph; because the wedge angle was back-calculated from postoperative radiographs, zero surgical error was assumed.

**Appendix Table A2.** Sensitivity analysis (9–15 months follow-up subgroup; n = 57 overall [80.3% of the total cohort]): Comparison of actual Post-WBLR and simulated Post-WBLR derived from standing, supine, and valgus-stress whole-leg radiographs^a^

|  | Postop Valgus Subgroup (n=38) | | | | Postop Varus Subgroup (n=19) | | | |
| --- | --- | --- | --- | --- | --- | --- | --- | --- |
|  | Mean difference^c^ | *P* value^d^ | RMSE | P value^e^ | Mean difference^c^ | *P* value^d^ | RMSE | P  value^e^ |
| Simulated  Post-WBLR_standing_^b^ | -9.6 ± 7.7 [-12.2, -7.1] | <.001 | 12.3 | <.001 | -6.4 ± 6.8 [-9.7, -3.1] | <.001 | 9.2 | .441 |
| Simulated  Post-WBLR_supine_^b^ | -0.7 ± 6.8 [-2.9, 1.6] | .538 | 6.8 | - | 5.0 ± 7.1 [1.6, 8.5] | .006 | 8.6 | - |
| Simulated  Post-WBLR_valgus_^b^ | 14.7 ± 7.1 [12.4, 17.0] | <.001 | 16.3 | <.001 | 25.2 ± 16.4 [17.3, 33.1] | <.001 | 29.8 | < .001 |

^a^Post-WBLR, postoperative weight-bearing line ratio; RMSE, root mean square error; Postop, postoperative; CI, confidence interval.

^b^Defined and illustrated in Figure 1.

^c^Mean ± standard deviation [95% CI].

^d^Comparison between the simulated and the actual Post-WBLR (paired t-test).

^e^Comparison of RMSE versus the supine-based simulation (Wilcoxon signed-rank test on squared errors).

**Appendix Table A3.** Univariable linear regression analysis identifying predictors of postoperative WBLR in the postoperative valgus group^a^

| Dependent variable | Explicative variables | Coefficient | P value |
| --- | --- | --- | --- |
| Actual Post-WBLR | Age | 0.01 | .913 |
|  | Sex | 6.34 | **.007** |
|  | BMI | 0.32 | .258 |
|  | K-L grade | 4.25 | .192 |
|  | Preop standing WBLR | 0.07 | .477 |
|  | Preop supine WBLR | 0.18 | .183 |
|  | Preop valgus-stress WBLR | 0.20 | **.053** |
|  | Preop standing aHKAA | 0.47 | .305 |
|  | Preop standing mLDFA | -0.36 | .556 |
|  | Preop standing JLOA | 0.03 | .941 |
|  | Preop standing MPTA | 0.25 | .571 |
|  | Preop standing JLCA | 0.20 | .724 |
|  | Preop supine JLCA | -0.06 | .921 |
|  | Preop valgus-stress JLCA | 0.30 | .620 |
|  | Preop ∆JLCA_standing-supine_ | 0.35 | .600 |
|  | Preop ∆JLCA_supine-valgus_ | -0.37 | .551 |
|  | Back-calculated wedge angle | 1.11 | **.012** |
|  | Simulated Post-WBLR_standing_ | 0.45 | **< .001** |
|  | Simulated Post-WBLR_supine_ | 0.53 | **< .001** |
|  | Simulated Post-WBLR_valgus_ | 0.53 | **< .001** |

^a^WBLR, weight-bearing line ratio; Post, postoperative; BMI, body mass index; K-L, Kellgren-Lawrence; aHKAA, arithmetic hip-knee-ankle ankle; mLDFA, mechanical lateral distal femoral angle; JLOA, joint line obliquity angle; MPTA, medial proximal tibial angle; JLCA, joint line convergence angle.

**Appendix Table A4.** AUC values for JLCA-related parameters in predicting planning outlier of supine-based simulation at varying thresholds of absolute prediction error in the postoperative valgus group^a^

| Parameters\Outlier threshold | 8% | 9% | 10% |
| --- | --- | --- | --- |
| JLCA_standing_ | 0.644 | 0.624 | 0.587 |
| JLCA_supine_ | 0.578 | 0.505 | 0.532 |
| JLCA_valgus-stress_ | 0.519 | 0.456 | 0.528 |
| JLCA_standing-supine_ | 0.664 | **0.725** | 0.675 |
| JLCA_standing-valgus_ | 0.645 | 0.674 | 0.613 |
| JLCA_supine-valgus_ | 0.615 | 0.617 | 0.560 |

^a^AUC, area under the receiver operating characteristic curve; JLCA, joint line convergence angle; WBLR, weight-bearing line ratio. Planning outlier was defined as an absolute discrepancy between the actual postoperative WBLR and the simulated WBLR derived from supine radiograph exceeding the specified thresholds (8%, 9%, and 10%).
